# The Perceived Power and Powerlessness in School Health Nurses’ Mental Health Promotion Practices: A Synthesis of Qualitative Studies

**DOI:** 10.1177/10598405241241212

**Published:** 2024-04-11

**Authors:** Nina Flodin, Stina Lejtzen, Hrafnhildur Gunnarsdóttir

**Affiliations:** 1 Department of Health Sciences, 42749 University West, Trollhättan, Sweden; 2 Lindholmens Tekniska Gymnasium, The Educational Administration, City of Gothenburg, Sweden; 3 Children's and Adolescent's Medical Services, Region Västra Götaland, Sweden

**Keywords:** School nursing, meta-synthesis, qualitative research, mental health, health promotion

## Abstract

Schools are important arenas for mental health promotion initiatives. School nurses have the opportunity and ability to support and promote students’ mental health, but their role and practices have been perceived as somewhat unclear. Therefore, the aim of this study was to explore school nurses’ mental health promotion practices. A total of 12 scientific studies were synthesized through a meta-ethnographic approach. The overarching results of the synthesis show that school nurses’ mental health promotion practices are largely about balancing and combining the students’ needs with different professional perspectives, competencies, and conditions. The school nurses perceived that they had the power to influence their practices through a variety of ways, highlighting the importance of letting the students’ needs guide the practices. Yet, at the same time they described feelings of powerlessness because of the different organizational structures that were hindering their mental health promotion practices.

## Background

A focus on the mental health of children and young people is vital, the foundations for positive development and good health are laid during childhood and adolescence (United Nations Children's Fund [UNICEF], 2021). The World Health Organization (WHO) defined mental health as “a state of well-being in which each individual can realize his own potential, cope with ordinary stresses, work productively and contribute to the society in which he lives” (WHO, 2001, p. 12). WHO (2001) further stated that mental health is about more than just the absence of mental illness or disability. Determinants of mental health are social, psychological, and biological, e.g., family conditions, emotional skills, substance use, and genetics. This means that some individuals and individuals living in certain social conditions can be more vulnerable to mental health problems than others. Further, adolescent wellbeing has been defined as having the “support, confidence, and resources to thrive in contexts of secure and healthy relationships, realizing their full potential and rights” (Ross et al., 2020, p. 473), which also implies that mental health is more than absence of illness or disability.

At its core, health promotion is about creating conditions that enable individuals to improve and maintain their health. Health is created where people live, love, and learn (WHO, 1986), so health promotion interventions need to be implemented in the contexts where people spend much of their time. Children and adolescents spend most of their waking time at school. Thus, the context of the school (Leger et al., 2022) and the role of school nurses (Whitehead, 2006) are significant in promoting the mental health of children and adolescents. By forming trusting and supportive relationships with students and being alert to changes in students’ mental health, school nurses can contribute to successful school performance and positive mental health (Bohnenkamp et al., 2015). Further, to promote mental health, collaboration is required between all professions in the school, health agencies in the community, municipalities, families, and students (Cowan et al., 2013).

School nurses’ responsibilities include preserving and promoting the health of schoolchildren and noticing unfavorable conditions in the psychosocial environment of students. The role also includes paying attention to and helping students who have physical and mental health problems. When the students visit the school nurse spontaneously, not having to schedule an appointment, it implies easy accessibility for children and young people to receive support, and gives school nurses opportunities to reach out to all students (Skundberg-Kletthagen & Larsen Moen, 2017). School performance and mental health affect each other bidirectionally, where mental health problems can make schoolwork more difficult, and difficulties performing in school can contribute to mental health problems (DeSocio & Hootman, 2004). School nurses are described as gateway professionals for mental health services in schools (Cowell, 2019), but their role and practice in mental illness and suicide prevention, as well as in mental health promotion, has been found to be somewhat unclear and challenging (Tyndall et al., 2023; Jönsson et al., 2019; Bohnenkamp et al., 2015)

When it comes to understanding and planning health promotion practices, a salutogenic approach is useful (Lindström & Eriksson, 2005), since it focuses on individuals’ resources and health instead of risk factors and disease. According to the salutogenic model of health, life is naturally full of challenges or stressors, and health is determined by an individual's ability to cope with these stressors (Antonovsky, 1979, 1996). In turn, an individual's ability to cope with stressors is based on the individual's sense of coherence and access to resistance resources (Antonovsky, 1979, 1996). A sense of coherence is based on three factors; 1) meaningfulness, which implies a sense of meaningfulness with being able to handle one's situation; 2) comprehensibility, which implies an urge to understand one's situation; 3) manageability, which implies the experience of having sufficient resources to handle one's situation. The resistance resources include factors such as personal characteristics, access and quality of social networks and relations, as well as social conditions (Antonovsky, 1979). In a school context, students’ sense of coherence is related to factors such as feeling pressed by schoolwork, social support from peers, and expectations (Natvig et al., 2006)

Promoting the mental health of children and young people is crucial for their wellbeing, both now and in the future, and schools are highly relevant arenas for mental health promotion initiatives. Previous research has highlighted that school nurses, through their competence and role, have unique opportunities and abilities to support and promote students’ mental health. However, there is a need for further research regarding their practices when promoting mental health. Thus, the aim of the study is to explore school nurses’ experiences of mental health promotion practices.

## Method

A meta-ethnographic study was conducted according to Noblit and Hare's method of meta-ethnographic synthesis (1988). Metaethnography is an interpretive method that, through interpretation, comparison, and analysis of qualitative studies, aims to form new theories that gain greater significance than a compilation of the results of individual studies (France et al., 2019), and it has been proven possible to use the method when synthesizing qualitative studies in order to form new theories (Britten et al., 2002). To a great extent, the synthesis is based on a Nordic perspective, as the majority of the selected articles present results from studies carried out in the Nordic countries, although Australia, England, and Portugal are also represented.

## Data Collection

Data in the form of scientific articles were collected via databases such as CINAHL, PubMed, PsycArticles, and PsycInfo, which contain accessible research in subject areas that are relevant to the purpose of the study. In CINAHL, PsycArticles, and PsycInfo, the keywords “school nurse,” “mental health,” “psychological well-being,” “health promotion” and “school health services” were used for searching. In PubMed, “school nursing” was used instead of “school nurse” because that is the term used in PubMed. Otherwise, the same keywords were used in PubMed as in the other databases. Table 1 provides an overview of the search procedure in the respective database. In addition to data collection via databases, the reference lists of the included articles were also checked manually to potentially find additional relevant articles.

**Table 1.** Search Strategy and Results.

Database	Search strategy	Results
CINAHL (EBSCO host)	Expanders - Apply equivalent subjects Search modes - Find all my search terms	
	S1: SU school nurse	21,084
	S2: SU mental health	205,114
	S3: SU psychological well-being	41,333
	S4: SU health promotion	95,335
	S5: SU school health services	28,613
	S6: S2 OR S3	236,871
	S7: S1 AND S4 AND S5 AND S6	77
	S8: S1 AND S4 AND S5 AND S6 Limiters: Peer Reviewed, Published Date 2012 - 2022 Narrow by Language: English	**42**
PsycInfo & PsycArticles (EBSCO host)	Expanders - Apply equivalent subjects Search modes - Find all my search terms	
	S1: SU school nurse	29,106
	S2: SU mental health	748,066
	S3: SU psychological well-being	70,794
	S4: SU health promotion	113,828
	S5: SU school health services	142,936
	S6: S2 OR S3	777,756
	S7: S1 AND S4 AND S5 AND S6	457
	S8: S1 AND S4 AND S5 AND S6 Limiters: Peer Reviewed, Published Date 2012 - 2022 Narrow by Language: English	**212**
PubMed	S1: School nursing [MeSH Terms]	5,528
	S2: Mental health [MeSH Terms]	50,965
	S3: Psychological well-being [MeSH Terms]	34,035
	S4: Health promotion [MeSH Terms]	82,569
	S5: School health services [MeSH Terms]	24,055
	S6: S1 OR S5	24,055
	S7: S2 OR S3	69,182
	S8: S4 AND S6 AND S7	100
	S9: S4 AND S6 AND S7 Filters: from 2012–2022, English	**59**

## Selection

**Figure 1.**
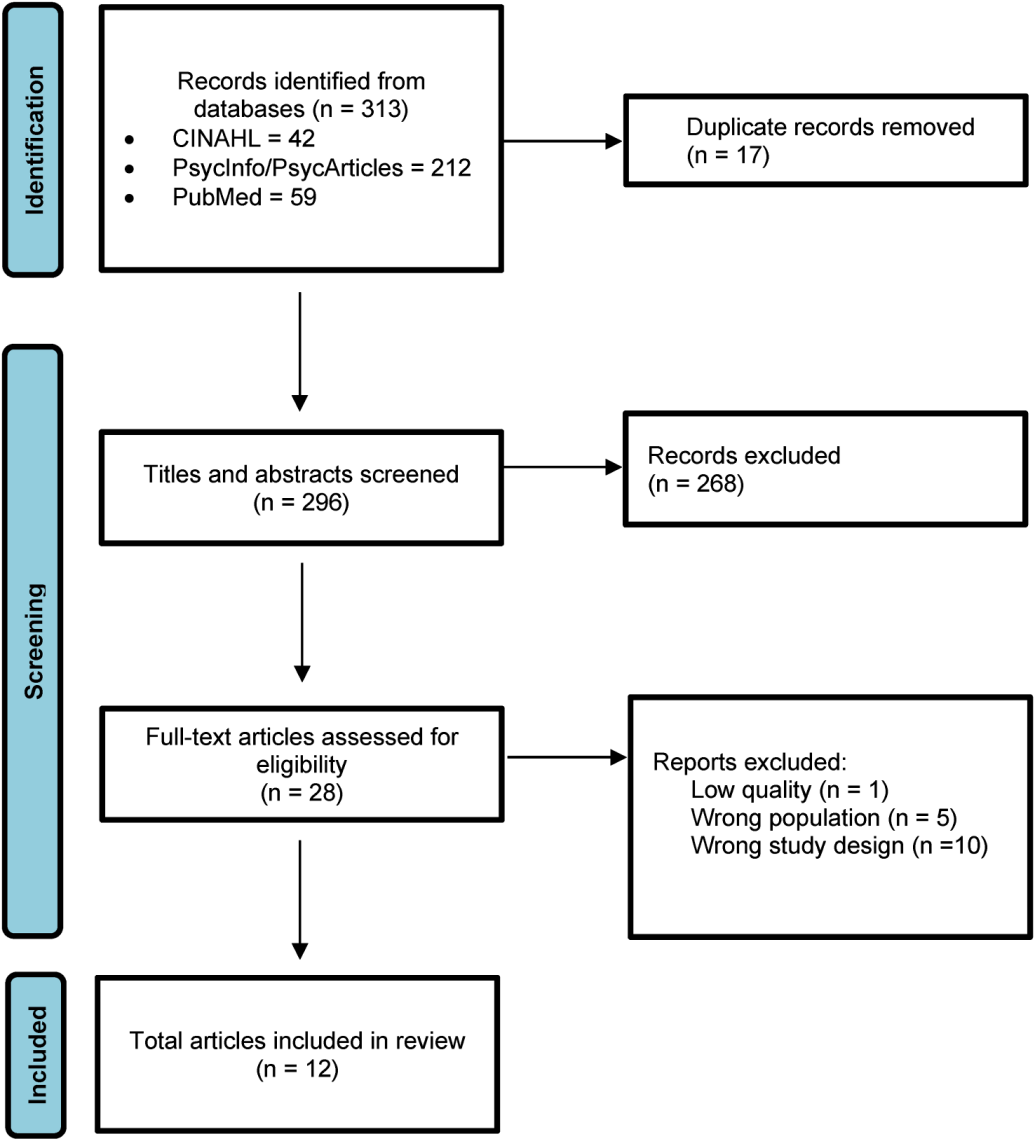
PRISMA flow chart illustrating literature search (Page et al., 2021).

Inclusion criteria were studies that focus on school nurses, children and young people, mental health, and health promotion, and qualitative or mixed research methodology. Peer-reviewed articles, published between 2012–2022 in English were included. A total of 313 articles were obtained through the searches and downloaded to the computer program Rayyan, which is a tool for systematizing articles into literature reviews (Ouzzani et al., 2016). See the flow chart in Figure 1 for the data selection. After duplicates were removed, 296 articles remained. Each article was reviewed based on the title and abstract and categorized using the include, exclude, and maybe features. The articles were reviewed individually by two of the authors and the decisions were compared. A discussion was held on the articles where there was a conflict between inclusion/exclusion until an agreement was reached. We also reviewed the title and abstract of the articles that were found manually through the reference lists of the included articles. A total of 269 articles were excluded because the study population, study design, or outcome did not meet the aim of the synthesis. From the manual search of included studies’ reference lists, one article was added for full-text review. A total of 28 articles were read in full-text and quality-reviewed according to a checklist designed for the quality review of qualitative studies (Swedish Agency for Health Technology Assessment and Assessment of Social Services, 2020). The purpose of the quality review was to assess the credibility, reliability, confirmability, and transferability of the studies (Polit & Beck, 2021). After the quality review, 16 articles were excluded because the study population or study design was not applicable for the purpose of the synthesis. In total, 12 articles were included in the synthesis (see Table 2).

**Table 2.** Overview of the Included Articles.

Author (year), country	Title	Purpose	Context, participants	Age group in focus	Method	Results	Key findings
Anttila et al. (2020), Finland	School Nurseś Perceptions, Learning Needs and Developmental Suggestions for Mental Health Promotion: Focus Group Interviews	School nurseś perceptions, learning needs and developmental suggestions for mental health promotion in students.	School nurses from junior and senior high schools in a larger city in southern Finland. N = 12	Students, 12–19 years old.	Focus group interviews. Qualitative content analysis.	Three main themes: school nurses’ perceptions of health promotion for young people's mental health in school, school nurses’ need for knowledge, skills and support in school and school nurses’ suggestions for the development of future health promotion for young people's mental health.	Cooperation. Families’ needs and experiences. Management/organization. School nurses’ experiences.
Dahlen Granrud et al. (2019), Norway	Public health nurseś perceptions of interprofessional collaboration related to adolescentś mental health problems in secondary schools: A phenomeno- graphic study	To describe school nurses’ perceptions of interprofessional collaboration related to adolescent mental health problems in secondary schools in Norway.	School nurses from secondary schools in sixteen different municipalities in Norway. N = 18	Students, 13–16 years old.	Individual interviews. Phenomenographic method of analysis.	Three descriptive categories: the formal structure has an effect on interprofessional collaborations, the school nurse is an important, but not always obvious, partner in interprofessional collaborations and the primary actors in the collaboration are the teachers.	Cooperation. Management/organization. School nurses’ experiences.
Garmy et al., (2015), Sweden	A qualitative study exploring adolescentś experiences with a school-based mental health program	To explore adolescents experiences of a school-based mental health program.	Adolescents from six schools in four different municipalities in southern Sweden N = 89	Adolescents, 13–15 years old.	Focus group interviews. Qualitative content analysis.	Three main categories: interpersonal strategies, interpersonal awareness, and structural constraints.	Adolescents needs and experiences
Laholt et al. (2019), Norway.	How to use visual methods to promote health among adolescents: A qualitative study of school nursing	To investigate how school nurses can use visual methods to promote health in adolescents.	Nurses from youth clinics and schools in a town in northern Norway. N = 40	Adolescents.	Focus group- discussions, observations, workshops. Systematic text- condensation	Three themes: photovoice gave adolescents a more active role in health promotion, photovoice's usefulness in focusing on public health issues, and benefits and challenges in implementing new methods in the school nurse's work.	School nurses’ experiences. Adolescentś needs and experiences.
Larsson et al. (2014), Sweden.	Striving to Make a Positive Difference: School Nurseś Experiences of Promoting the Health and Well-Being of Adolescent Girls	To illuminate school nurses’ experiences of promoting health and well-being in adolescent girls.	School nurses from junior high and high schools in various small and medium-sized cities in municipalities in western Sweden. N = 17	Adolescents (girls), 12–20 years old.	Individual interviews and group interviews. Phenomenographic method of analysis	Six components: striving to establish trusting relationships, helping the girls to gain insight into their situation, supporting a healthy rhythm of life, being reliable when the girls have serious problems, trying to de-dramatize values and attitudes, and collaborating for supporting health and learning.	School nurses experiences
McCallister et al. (2019), Australia.	Evaluation of a professional development experience designed to equip school support staff with skills to facilitate youth mental health promotion	To report the results of a training course for facilitators of a mental health program for high school students.	Mediators from secondary schools in central Queensland in Australia who were involved in a mental health promotion program. The mediators were, among other things, teachers, study path leaders and school nurses. N = 27 (School nurses N = 5)	Adolescents, 12–18 years old.	Mixed method, questionnaires and evaluation survey with qualitative questions. Thematic analysis of qualitative data.	Four themes emerged from the thematic analysis of the qualitative data: learning needed, things that worked well, criticisms and further improvements.	School nurses experiences.
Oliveira Costa et al. (2022), Portugal	A qualitative study exploring adolescentś perspective about Mental Health First Aid Training Programmes promoted by nurses in upper secondary schools	To examine adolescentś perspectives on a mental health program delivered by school nurses.	Adolescents from a secondary school in Portugal. N = 12	Adolescents, 15–18 years old.	Individual telephone interviews. Qualitative content analysis.	Three main areas: the relevance of the training programs, the content of the training programs and the intervention methods of the training programs.	Adolescents needs and experiences.
Onnela et al. (2014), Finland	Mental health promotion in comprehensive schools	To investigate current methods for health promotion work for mental health in elementary school and to develop a professional model for interventions related to mental health through the use of an action research process.	Principals, teachers, guidance counselors, counselors, school nurses, school psychologists, interest groups, students and parents from eight primary schools in the city of Oulo, Finland.	Children and adolescents, 7–16 years old.	Workshops, action research. Content analysis.	School mental health interventions: school-wide events, knowledge sharing, low-threshold sites, school staff training, selective interventions, classroom interventions, groups, indicated interventions and support for students and families.	Families’ needs and experiences. Children's and adolescents needs and experiences.
Pryjmachuk et al. (2012), UK	School nurseś perspectives on managing mental health problems in children and young people	To explore school nurses’ images of young people's mental health problems and their potential to engage in youth mental health work.	School nurses from four school nurse teams in two UK cities. N = 33	Children and young people.	Focus group interviews. “Framework” analysis method to bring out different themes.	Four main themes: child and adolescent mental health, organizational issues, barriers to working with mental health and facilitating factors for working with mental health.	Cooperation. Families’ needs and experiences. Management/organization. School nurses’ experiences.
Putkuri et al. (2021), Finland	Good interaction skills are not enough – competency in mental health issues in child health clinics and school health services	To describe the nursing skills required to work with mental health problems in childcare centers and in schools.	Nurses from childcare centers and schools in southern and eastern Finland. N = 24	Children and adolescents	Focus group interviews. Content analysis.	Main competences that emerged were: intuitive and interpersonal competence and theoretical and evidence-based competence.	Families’ needs and experiences. School nurses’ experiences.
Reuterswärd and Hylander (2017), Sweden	Shared responsibility: school nurseś experience of collaborating in school-based interprofessional teams	To describe how Swedish school nurses experience their work and their collaboration within interprofessional teams.	School nurses from primary and secondary schools in four different municipalities around Stockholm. N = 25	Children and adolescents, 6 - 18 years old.	Focus group interviews. Content analysis.	Three domains: expectations of the school nurse's role, the school nurse's contribution to student health and well-being, and the school nurse's collaboration within the student health team.	Cooperation. Management/organization. School nurses’ experiences.
Savolainen et al. (2021), Finland	Public health nurseś perceptions on promotive and risk factors for childreńs mental health: A qualitative interview study	To describe health-promoting factors as well as risk factors for children's mental health on the individual, interpersonal, organizational, municipal and political levels of the socio-ecological environment.	Nurses from childcare centers and schools in eight different Finnish municipalities. N = 23	Children and adolescents.	Semi-structured interviews. Content analysis	Three categories emerged at the individual and interpersonal levels: the child's overall well-being, factors within the family and supportive networks and other relationships. Five categories emerged at the organizational, municipal and political levels: activities targeting children, parents and families in primary care, activities in schools and kindergartens, activities and conditions in the sectors environment, construction, culture and sports, municipal interventions for mental health and structures of mental health promotion.	Families’ needs and experiences. School nurses’ experiences.

## Analysis

We applied a meta-ethnographic approach based on Noblit and Hare's (1988) method for synthesizing qualitative studies. The meta-ethnographic synthesis was conducted in seven interactive steps according to Noblit & Hare's (1988) method by two of the authors, while the third author validated the analysis by reading and discussing the results regularly throughout the process. Step 1 involved choosing the area to be investigated and whether a qualitative method is suitable for investigating the topic. As the purpose of this synthesis was to explore school nurses’ perspectives and experiences, a qualitative method was considered compatible. In Step 2, studies were selected in relation to the purpose of the synthesis and the interests of the intended readership. In this step, studies were collected via four different databases and then reviewed based on the inclusion criteria mentioned in the selection. Step 3 comprised a thorough and repeated reading of the studies with the aim of exploring the content through interpretation and drawing attention to key findings and themes. The results of the included studies were documented in a table so patterns were easily discerned. This was followed by repeated reading of the results of the included studies and identifying key findings that were significant for the purpose of the synthesis. Through Step 4, connections are sought between the studies by creating a clear overview of key findings and concepts. The key findings that were identified included children's and adolescents’ needs and experiences, school nurses’ experiences, families’ needs and experiences, cooperation and management/ organization. The key findings and their implications from each individual study were written down as a consolidated text, repeatedly read, and discussed. Step 5 involved transferring and interpreting the results of the studies by drawing parallels and/or finding contrasts within and between studies. By summarizing the content of the key findings in a joined text, the individual results of the studies could be transferred and jointly interpreted based on central meanings that were relevant to the purpose of the synthesis as proposed by Noblit and Hare (1988). The key findings were then compared with the results of the original studies to ensure that the essence of the studies had not been lost. After textual review, the key findings were reduced and coded based on common meanings. In Step 6, the product of the interpretations was synthesized into a new whole that exceeds a compilation of the individual studies, while Step 7 included the summation of results and the conveyance of the synthesis. In this step, the codes were interpreted, compared, and divided into five sub-themes that constituted two overarching themes. Table 3 shows examples of the interpretation procedure. The focus of the synthesis is on a mutual translation of included studies, as there were so few conflicting findings. We worked together through the whole process, and the different findings got different text colors to more easily differentiate and connect similarities.

**Table 3.** Examples of Interpretation and Synthesizing.

Key findings	Code	Subtheme	Theme
Children's and adolescents needs and experiences	Active role Positive approach Communication	To strengthen the students empowerment and sense of participation	Letting the students’ needs lead the way
School nurses experiences	Competence Intuition Encourage and support	To actively listen and see the individual	
Families’ needs and experiences	To include families Knowledge about family relationships	To create positive relationships with families	
Cooperation	Dysfunctional cooperation Confidentiality as an obstacle Desire for community	Feelings of loneliness and exclusion within the school's interprofessional collaboration	Consequences of working in a interprofessional context
Management/ Organization	Lack of support from school management A wish for a change Attitude from colleagues	Experiencing powerlessness in the universal mental health promotion practices	

## Results

A total of 12 qualitative studies were included in the meta-ethnographic synthesis. Table 4 includes an overview of themes and subthemes represented in the included articles. The repeated reading, coding, and interpretation of the studies resulted in five subthemes that formed two main themes: Letting the students’ needs lead the way and the consequences of working in an interprofessional context. An overview of the results of the synthesis and the identified themes and subthemes is shown in Table 5.

**Table 4.** Overview of Themes and Subthemes Represented in Included Articles.

References	Themes and subthemes				
	Letting the students’ needs lead the way			Consequences of working in a interprofessional context	
	To strengthen the students empowerment and sense of participation	To actively listen and see the individual	To create positive relationships with families	Feelings of loneliness and exclusion for school nurses within the school's interprofessional collaboration	Experiencing powerlessness in the universal mental health promotion practices
Anttila et al. (2020) Dahlen Granrud et al. (2019) Garmy et al., (2015) Laholt et al. (2019) Larsson et al. (2014) McCallister et al. (2019) Oliveira Costa et al. (2022) Onnela et al. (2014) Pryjmachuk et al. (2012) Putkuri et al. (2021) Reuterswärd and Hylander (2017) Savolainen et al. (2021)	X X X X X X	X X X X	X X X X X	X X X X	X X X X

**Table 5.** Results of the Synthesis With Themes and Subthemes.

Results of the synthesis	Theme	Subtheme
Balancing and combining the students’ needs with different professional perspectives, competencies, and conditions	Letting the students’ needs lead the way	To strengthen the students empowerment and sense of participation
		To actively listen and see the individual
		To create positive relationships with families
	Consequences of working in an interprofessional context	Feelings of loneliness and exclusion within the school's interprofessional collaboration
		Experiencing powerlessness in the universal mental health promotion practices

## Balancing and Combining the Students’ Needs with Different Professional Perspectives, Competencies, and Conditions

The overarching results of the synthesis show that school nurses’ mental health promotion practices are largely about balancing and combining the students’ needs with different professional perspectives, competencies, and conditions. These results consist of the two themes mentioned above. The first theme – letting the students’ needs lead the way – captures aspects of school nurses’ competence and roles in promoting mental health in children and adolescents on a micro level; that is, in their interaction with the individual student or small groups of students, where the importance of letting the students’ needs guide the work was found to be crucial. The latter theme – consequences of working in an interprofessional context – implies various obstacles that the school nurses experienced on the organizational and managerial levels. It emerged that macro-level systems, such as structures in society and in the school organization, were perceived to impede school nurses’ universal health promotion practices and led to feelings of loneliness and powerlessness. The two themes, with associated sub-themes, are described below.

## Letting the Students’ Needs Lead the way

On a micro level, the school nurses were able to facilitate the promotion of mental health in children and adolescents in several different ways. Through their role, the school nurses strove to increase students’ empowerment and sense of participation, which were seen as important components of mental health promotion practices. The school nurses’ abilities to listen and see the individual were seen as important qualities. The creation of positive relationships with families and guardians was considered crucial, even though it was often experienced as a challenge, with several aggravating obstacles. Thus, letting the students’ needs lead the way in the mental health promotion practices proved to be a key.

### To Strengthen the Students’ Empowerment and Sense of Participation

An effort to strengthen the students’ empowerment and sense of participation was central in the school nurses’ practices when promoting mental health (Anttila et al., 2020; McCallister et al., 2019; Oliveira Costa et al., 2022; Onnela et al., 2014). The school nurses described how the work could be done both as group activities for all students and more targeted ones, such as to boys or girls only. Physical meetings were generally preferred over digital ones by both the school nurses and the students (Anttila et al., 2020). It was found that the physical meetings enabled the students to maintain concentration and attention for a longer period of time than the digital meetings (Oliveira Costa et al., 2022).

Classroom interventions carried out by school nurses were described as feasible ways to increase knowledge about mental health, which was something the students felt they needed and appreciated. It was found to increase their mental health literacy, enabling them to take a more active role regarding their mental health (Oliveira Costa et al., 2022). Through group activities, the young people experienced an increased understanding of other people, as well as a reduced feeling of loneliness. They highlighted the desire for less focus to be placed on negative parts and performance, and more on communication, which they believed would give the activities more depth. They suggested that it could be carried out through practical exercises and role plays (Garmy‌ et al., 2015). Classroom interventions were also found to promote mutual support within the group, providing a collective sense of coherence and of belonging. They provided methods and knowledge to students to increase and maintain positive mental health, and it was important to adopt an approach that aimed to empower them. In the case of interventions on a selective level, it was pinpointed that the content could also be tailored to the students’ level of development. It was seen as an advantage in the development of new methods to promote mental health within the school context when school staff, parents, students, and healthcare staff were all regarded as mutually important participants who worked together (Onnela et al., 2014).

The school nurses expressed a desire to act as facilitators to help the students find solutions themselves and propose tools to help themselves. The nurses described how promotion work included a focus on dialogue and student-centered content rather than one-way information about health-related issues. Students should be regarded as experts in their own lives, which was considered to increase the opportunity for inclusion, connection, participation, dialogue, and sharing (McCallister et al., 2019). Using visual methods, such as photos taken by the students, when having conversations about health issues has been found to give the students a more active role in the conversation and increase their control over what topics should be covered in the conversations (Laholt et al., 2019).

### To Actively Listen and see the Individual

The ability to actively listen was seen as an important component of the school nurses’ mental health promotion practices (Pryjmachuk et al., 2012; Savolainen et al., 2021). The school nurses described how they sometimes lacked knowledge, as well as being afraid of saying or doing something wrong when working with the mental health of children and adolescents. Thus, the ability to listen and to give the student time was perceived as fundamental (Pryjmachuk et al., 2012).

The school nurses also emphasized being aware of and taking their students’ individual characteristics into account since these affect how they experience their surroundings, and all children need to feel safe, trusted, and accepted (Savolainen et al., 2021). It was highlighted that, when working with mental health promotion, it is important to affirm and focus on students’ self-esteem and to take an optimistic perspective on situations. This could be done by supporting students to process emotions by listening to music, drawing, writing, and helping them put names to their emotions (Putkuri et al., 2021). One study, which focused on girls only, highlighted an aspiration to establish trusting relationships, as well as a desire to help girls achieve insights into their situation and feelings. It turned out to be important to be reliable when girls have serious problems, as well as trying to de-dramatize challenging attitudes and ideals to achieve well-being (Larsson et al., 2014).

### To Create Positive Relationships with Families

Collaboration with parents or other guardians was highlighted as an important part of the practices when promoting the mental health of the students (Anttila et al., 2020; Putkuri et al., 2021; Savolainen et al., 2021). The parents were considered to have great influence over the lives of children and adolescents, and the school nurses requested more resources to better engage guardians in the practices of promoting mental health (Anttila et al., 2020). The nurses highlighted the cooperation with parents and family as sometimes being crucial because a good flow in life at home, with a regular circadian rhythm and balance, are fundamental prerequisites for psychological well-being in children and adolescents. The school nurses described how safe family and home conditions were perceived as protecting children in challenging situations (Savolainen et al., 2021). Whether parents were included in the health dialogues depended on the student's age and preferences. Among younger students, such as those at a primary school level, it was found especially important, while for older students, it could be important to be able to choose whom to involve in the conversations; this was sometimes another adult they trusted (Onnela et al., 2014).

Although it was considered important to include guardians, this was also found to be challenging. The school nurses described how the advice given to parents was rarely followed and that parents sometimes had difficulty seeing their own part and responsibility in the students’ situations. Parents could also be demanding, e.g., demanding rapid improvements in their child's mood or behavior, even though such changes take time (Pryjmachuk et al., 2012). The school nurses also stated that through parental contact they could learn about health issues and problems in the families and, because intrafamilial relationships are of central importance for the psychological well-being, parent contact was found supporting for their health promotion practices (Putkuri et al., 2021).

## Consequences of Working in an Interprofessional Context

The school nurses reported that working from different professional perspectives compared to school management and other school staff could have consequences. The school's organizational structures were described as challenging for the school nurses’ mental health promotion practices. The school nurses experienced feelings of loneliness and exclusion within the school's interprofessional collaboration. Due to a lack of cooperation, promoting mental health was perceived as challenging. Organizational obstacles were perceived as leading to negative impacts on students’ access to interventions to promote mental health, as well as a feeling of powerlessness among the school nurses. Both general social structures and structural systems within the school were found to be challenging for the promotion of mental health on a universal level. The school nurses experienced difficulties in changing and influencing macro-level structures, such as the number of students and sufficient staffing of school nurses, which had negative effects on the promotion practices.

### Feelings of Loneliness and Exclusion Within the School's Interprofessional Collaboration

A lack of cooperation with other professionals in the school was perceived to contribute to feelings of loneliness and exclusion (Dahlen Granrud et al., 2019). As an individual professional category within the school, the school nurses reported that they did not have the collegial support that the teachers had and that they sometimes were not included in collaboration regarding the students’ mental health. A contributing factor to a feeling of exclusion was the fact that the school nurses often worked at more than one school simultaneously. As a result, they felt divided and left out of the professional community at the various schools.

The school nurses described that it was up to the teachers if they wanted to start a collaboration, making them dependent on the goodwill of the teachers regarding joint mental health promotion practices. Teachers, who are newer to the profession were depicted as more frequent and willing collaborators, compared to the more senior teachers. More senior teachers were said to have a more negative attitude towards the role and practices of the school nurses and handled the students’ psychological well-being individually (Dahlen Granrud et al., 2019). Collaboration was linked to teachers’ individual attitudes regarding the practices of school nurses and whether there was a willingness to form a professional relationship (Pryjmachuk et al., 2012). The school nurses also described being isolated and their work was often misinterpreted (Anttila et al., 2020; Dahlen Granrud et al., 2019; Pryjmachuk et al., 2012; Reuterswärd & Hylander, 2017). Confidentiality that applies to school nurses was described as a potential obstacle to interprofessional collaboration (Dahlen Granrud et al., 2019; Reuterswärd & Hylander, 2017). It made it difficult for the school health nurses to discuss the health issues of specific students with other staff in the interprofessional meetings. In turn, the school nurses felt isolated and alone when it came to thoughts and information that they had related to students’ health issues (Reuterswärd & Hylander, 2017). The school nurses had a desire for collegial support. They sometimes needed to be able to discuss certain health-related issues quickly, and in the absence of these opportunities, the feeling of isolation within the professional role increased. School physicians, counselors, and special educators were seen as, in addition to teachers, being central to the school nurses’ collaboration and health promotion practices (Anttila et al., 2020; Reuterswärd & Hylander, 2017).

Facilitating factors for collaboration were whether the school nurses’ room was located next to, for example, the counselor's and special education teacher's rooms, and whether those staff had similar working hours. In this way, the feelings of loneliness and isolation could be reduced, and the work for the students could be improved (Reuterswärd & Hylander, 2017). The school nurses mentioned that their role was sometimes misunderstood by other professional categories in the school and that perceptions of the school nurses’ mental health promotion practices were diffuse (Pryjmachuk et al., 2012). Health promotion practices were said to be complicated to describe to people such as teachers and principals, and the school nurses had to repeatedly explain what this work entailed. Insufficient dialogue between the various professions in the school could lead to a lack of clarity regarding the role of school nurses, which also affected cooperation around the students. With functioning interprofessional information exchange and conversations, collaboration improved, and the school nurses became more frequently involved in school activities (Reuterswärd & Hylander, 2017).

### Experiencing Powerlessness in the Universal Mental Health Promotion Practices

A lack of time was seen as an obstacle for the school nurses to carry out universal health promotion practices in the school. The school nurses stated that they often shared their working time between several schools, and consequently found it difficult to manage work other than scheduled health visits and urgent matters. There was a desire among the school nurses to be able to spend more time on various forms of universal mental health promotion practices (Reuterswärd & Hylander, 2017). The school nurses’ desired to work more comprehensively with issues related to mental health and the desire to support a favorable and health-promoting culture at school was prominent (Anttila et al., 2020; Dahlen Granrud et al., 2019; Pryjmachuk et al., 2012; Reuterswärd & Hylander, 2017).

The school nurses described that by being limited in their availability at the school, they were occasionally excluded from collaboration with other professions. Because the work was often divided between several schools, they felt divided (Dahlen Granrud et al., 2019). The school nurses stated that they mostly worked with the promotion of mental health on an individual level rather than universal, because of the obstacles created by the lack of interprofessional collaboration. To work more universally, the nurses claimed that the whole school needed to be involved and the principal and teachers had to have a desire to focus on health promotion (Reuterswärd & Hylander, 2017). A well-functioning collaboration between the different professions at the school was highlighted when promoting students’ mental health through a holistic perspective based on different competencies (Dahlen Granrud et al., 2019).

Support from principals and school management was described as being central to school nurses’ opportunities to work with universal mental health-promoting initiatives. In the absence of support and lack of confirmation regarding the qualifications of school nurses, universal interventions for mental health in schools did not occur (Reuterswärd & Hylander, 2017). The school nurses also felt that their work was not always visible to principals and management and that financial or time investments into mental health promotion practices were lacking (Anttila et al., 2020).

## Discussion

The overarching results of the synthesis show that school nurses’ mental health promotion practices are largely about balancing and combining the students’ needs with different professional perspectives, competencies, and conditions. The results showed how the competence and role of school nurses were significant in making students’ needs visible, and it was important to let the needs act as a guide in the design of mental health promotion practices. The results also showed that school nurses’ health-promoting practices were influenced by organizational structures within school and society. Changes within these structures can provide improved conditions for successful mental health promotion practices within the school context and can also reduce school nurses’ feelings of loneliness and powerlessness.

The results of the present study have identified the central role of a safe home environment. School nurses’ collaboration with guardians can provide increased insights into and knowledge about the mental health of children and adolescents. This is in line with Skundberg- Kletthagen et al.'s (2017) study, which described how school nurses consider it important to be easily accessible to students in order to build a sense of security and trust. That study also described the importance of having insight into students’ home conditions and social relationships in order to be able to provide support in case of possible psychosocial and emotional difficulties (Skundberg-Kletthagen & Larsen Moen, 2017).

Health promotion is grounded in a salutogenic perspective on health, which shifts the focus from risk factors and disease to resource factors and health, which implies more solution-focused work that starts from the resources that are available (Lindström & Eriksson, 2005). The results of the present study showed that classroom interventions could be a successful way of working to promote students’ sense of participation and empowerment. It also appeared that safe family and home conditions could protect students in challenging situations, which can be interpreted as these conditions increased students’ resistance resources. Based on that point of view, the inclusion of guardians becomes an important part of mental health promotion work, which was also clearly identified in our results. Wolff and Ratner (1999) also believed that general resistance resources do not only consist of individual and material resources, but also relational ones such as social support. Previous research (Bowen et al., 1998; Natvig et al., 2006) has shown that support from classmates and teachers, as well as a sense of belonging and safety, are resilience resources that increase the sense of coherence. The sense of coherence, in turn, means a reduced risk of negative effects from stress and other pressures. We identified that classroom interventions were perceived as promoting mutual support within the group and increasing the sense of belonging, which in turn can be considered to increase students’ resistance resources.

Similarly, Cowan et al. (2013) described that a holistic perspective, through coordination among school, home, and society, with a focus on mental, emotional, physical, academic, and social factors, is needed to promote a positive school environment and mental health among children and adolescents.

In order to promote students’ mental health, it is necessary to have sufficient staffing of school nurses, school psychologists, and school counselors in relation to the number of students. Overly large student groups and a lack of time in the work of individual school nurses and other staff within the student health care services can lead to aggravating circumstances when implementing interventions at the group level and working for a positive health culture within the school (Cowan et al., 2013), which is also in line with our results.

Our results identified that school nurses sometimes experienced a lack of knowledge and a fear of making mistakes when working with mental health promotion of children and adolescents. According to Reuterswärd and Lagerström's (2010) study of the health promotion work of school nurses in Sweden, the school nurses based their work on municipal guidelines, which means that each municipality decides its own guidelines on how to handle certain situations. However, there was uncertainty about how health promotion practices would be carried out and it emerged that it was up to individual school nurses to reflect on and plan health promotion efforts, which is consistent with the results of the synthesis. As municipal guidelines differ in different regions and the school nurses stated that the health promotion practices are, in many ways, based on individual knowledge and working methods, the result can be an inequality in students’ conditions for healthy schooling and development based on differences in different municipalities.

There is a discrepancy in society between higher consumption of care related to mental illness in girls and the fact that boys and men are overrepresented in suicide statistics (Junuzovic et al., 2021; National Board of Health, 2019; UNICEF, 2021). In the included studies, the focus was mainly on all children and adolescents, with the exception of one study that focused on teenage girls. No studies focused on boys alone, despite the higher suicide risk. In the study that highlighted health promotion practices with a focus on teenage girls, it emerged that the school nurses strived to establish trusting relationships and emphasized the desire to help girls achieve insight into their situation and their feelings. The nurses believed that it was important to be reliable when girls had serious problems, as well as to try to de-dramatize values and attitudes in order to achieve well-being. However, these goals should be seen as applicable to all students, regardless of gender. According to the Discrimination Act (SFS, 2008, p. 567), school nurses’ efforts must promote gender equality. Connell (2015) described how ideals of femininity and masculinity are constructed in everyday life and emphasized the importance of institutional structures for this. For children and adolescents, school is an important context where gender ideals are created and perpetuated in different ways. Therefore, all professions within the school have a responsibility to counteract structures that reinforce destructive gender ideals and thus contribute to reducing destructive demands based on gender.

## Strengths and Limitations

The included studies were obtained through searches in four different databases. A check was also made of the reference lists of included articles to further broaden the basis for the synthesis. However, no articles from the reference lists met the inclusion criteria, which may indicate that the database searches were sufficiently exhaustive. The use of the computer program Rayyan in the review of the 313 articles located in the database searches facilitated the selection process by allowing inclusion and exclusion decisions to be made individually and then compared to ensure an understanding of the area and purpose of the study. When the decision was made to only include studies with qualitative or mixed research methodologies, a large number of articles were excluded, which can be seen as a weakness in terms of the relatively limited basis that constitutes the synthesis. Laholt et al.'s (2019) study, which investigated how school nurses can use visual methods to promote health in young people, was included in the synthesis even though it did not explicitly highlight mental health. However, it did draw attention to the fact that the methods can promote mental health in young people, and it also examined the importance of young people taking an active role in health promotion practices, which meant that it was considered relevant to the purpose and was therefore included. Reuterswärd and Hylander's (2017) study on school nurses’ experiences of their work and collaboration within the school was also included, even though the main purpose of the study did not exclusively focus on the mental health of children and adolescents. Nevertheless, the study was assessed as relevant for the synthesis because it provided an important insight into the role and conditions of school nurses. By including studies that were based on school nurses’ and children's and adolescents’ perceptions and experiences of mental health and health promotion practices, the synthesis can provide an in-depth and nuanced insight into school nurses’ practices and challenges.

The present synthesis is based on a systematic selection and analysis procedure, which is also explained in detail in the method section. The synthesis is based on a meta-ethnographic method, where every step in the process is reported. The included articles were all quality-reviewed according to the Swedish Agency for Health Technology Assessment and Assessment of Social Services’ (2020) template for reviewing qualitative articles. All of the included articles were initially read and interpreted individually and close to the text. The interpretations were then compared and discussed to ensure objectivity and that the information had not been distorted from its original meaning. Previous impressions and preconceptions that general interventions to promote mental health in children and adolescents at school were deficient and that guidelines for school nurses’ mental health promotion practices were unclear may have influenced the choice of topic and, to some extent, the results of the synthesis. However, discussion regarding pre-understanding has been continuously conducted during the work process to ensure as little influence as possible in the close-to-the-text interpretation of the results of included studies and to ensure that it is the participants’ perspective that is reflected.

The majority of the studies included in the present synthesis were conducted in a Nordic context. Also, all studies were conducted in the context of similar school systems, which indicates transferability to school nurses working in countries with similar conditions and school systems, but the transferability might be limited to school nurses working outside the Nordic countries. Given that all the articles were read individually and with the assurance that the results have been interpreted correctly, as well as the fact that a systematic data collection and analysis procedure has been carried out, the results of the synthesis are judged to be reliable. However, since the meta ethnographic synthesis is a method that is largely made up of the researchers’ own interpretations, part of the result in this type of study will always be influenced by the metaphors developed after the close-to-the-text interpretation. The synthesis can be useful for several different actors in addition to school health nurses, such as children and adolescents themselves, guardians, decision-makers, and relevant professionals.

## Conclusion

The overarching results of the synthesis show that school nurses’ mental health promotion practices are largely about balancing and combining students’ needs with different professional perspectives, competencies, and conditions. On a micro level, the school nurses perceived that they had the power to influence their work. They described a variety of ways to work with the students, individually and in small groups, and highlighted the importance of letting the students’ needs guide the mental health promotion practices. On a macro level, however, the school health nurses described feelings of powerlessness because organizational structures within schools hindered their opportunities for mental health promotion practices. Responsibility for overly large groups of students, insufficient interprofessional collaboration, and unclear guidelines were the main hindrances described. Therefore, school nurses must balance students’ needs and organizational conditions when promoting mental health.
